# Risk of Dementia After Smoking Cessation in Patients With Newly Diagnosed Atrial Fibrillation

**DOI:** 10.1001/jamanetworkopen.2022.17132

**Published:** 2022-06-15

**Authors:** Hui-Jin Lee, So-Ryoung Lee, Eue-Keun Choi, Sang-Hyun Park, Jae-Wook Chung, Jung-Min Choi, Min-Ju Han, Jin-Hyung Jung, Kyung-Do Han, Seil Oh, Gregory Y. H. Lip

**Affiliations:** 1 Department of Internal Medicine, Seoul National University Hospital, Seoul, South Korea; 2 Department of Internal Medicine, Seoul National University College of Medicine, Seoul, South Korea; 3 Department of Medical Statistics, College of Medicine, Catholic University of Korea, Seoul, South Korea; 4 Statistics and Actuarial Science, Soongsil University, Seoul, South Korea; 5 Liverpool Centre for Cardiovascular Science, University of Liverpool and Liverpool Chest and Heart Hospital, Liverpool, United Kingdom; 6 Department of Clinical Medicine, Aalborg University, Aalborg, Denmark

## Abstract

**Importance:**

Incident atrial fibrillation (AF) is associated with an increased risk of dementia. However, data on the association between smoking cessation after AF diagnosis and dementia risk are limited.

**Objective:**

To evaluate the association between changes in smoking status after AF diagnosis and dementia risk.

**Design, Setting, and Participants:**

This nationwide cohort study with 126 252 patients used data from the Korean National Health Insurance Service database, including patients who had a national health checkup examination within 2 years before and after AF diagnosis between January 1, 2010, and December 31, 2016. Based on their smoking status, participants were classified as never smokers, ex-smokers, quit smokers, and current smokers. Ex-smokers were defined as those who had quit smoking before the first examination and remained quit until the second examination. Patients who were current smokers at the first health examination but had quit smoking before the second examination were classed as quit smokers. The index date was the second health examination. Patients were followed up until dementia, death, or the study period ended (December 31, 2017), whichever occurred first. Data were analyzed from January 13, 2020, to March 29, 2022.

**Exposures:**

Smoking cessation after newly diagnosed AF.

**Main Outcomes and Measures:**

Dementia, including Alzheimer disease and vascular dementia, was the primary outcome. Cox proportional hazards regression model was used to estimate hazard ratios.

**Results:**

A total of 126 252 patients (mean [SD] age, 62.6 [12.0] years; 61.9% men) were included in the analysis. The mean (SD) CHA_2_DS_2_-VASc score, which measures the risk of ischemic stroke, was 2.7 (1.7). Smoking status of the total study population was as follows: 65 579 never smokers (51.9%), 34 670 ex-smokers (27.5%), 8919 quit smokers (7.1%), and 17 084 current smokers (13.5%). During a median of 3 years of follow-up, dementia occurred in 5925 patients (1.11 per 1000 person-years). After multivariable adjustment, the risk of quit smokers was significantly lower than that of current smokers (hazard ratio, 0.83 [95% CI, 0.72-0.95]).

**Conclusions and Relevance:**

The findings of this cohort study suggest that all types of smoking were associated with a significantly higher risk of dementia in patients with new-onset AF. Smoking cessation after AF diagnosis was associated with a lower risk of dementia than among current smokers. These findings may support promoting smoking cessation to reduce dementia risk in patients with new-onset AF.

## Key Points

**Question:**

Is smoking cessation in patients with newly diagnosed atrial fibrillation (AF) associated with a lower risk of incident dementia?

**Findings:**

In a cohort study of 126 252 patients with new-onset AF in South Korea, smoking cessation after an AF diagnosis was associated with a significantly lower risk of dementia than that of patients who were current smokers.

**Meaning:**

These findings suggest that smoking cessation could be more proactively promoted in patients with newly diagnosed AF to reduce the burden of AF-related dementia.

## Teaser

This cohort study assesses the association between quitting smoking and the risk of incident dementia among patients in South Korea with a new diagnosis of atrial fibrillation.

## Introduction

Atrial fibrillation (AF) is the most common cardiac arrhythmia, with an increasing prevalence in the elderly,^1^ conferring a high risk of adverse cardiovascular outcomes and health care costs.^2,3,4,5^ Cognitive dysfunction is prominent among patients with AF.^6^ Several observational studies and meta-analyses have shown that AF is an independent risk factor for cognitive decline or incident dementia, including Alzheimer disease and vascular dementia.^7,8,9^

Atrial fibrillation is also associated with multimorbidity; therefore, a more integrated or holistic care of AF has been advocated to improve clinical outcomes of patients with this condition.^10,11^ Specifically, recent guidelines have proposed the AF Better Care pathway, which indicates avoidance of stroke (anticoagulation), better symptom management (rhythm and rate control), and cardiovascular and comorbidity optimization, including lifestyle changes.^12^ Oral anticoagulation treatment and rhythm control play significant roles in lowering the risk of dementia in the population with AF as part of an integrated care approach.^13,14,15^

Nonetheless, limited data are available about the association of lifestyle modifications with the incidence of dementia in AF. It has been established that smoking is the most common risk factor for AF, stroke, and dementia.^16,17,18^ We hypothesized that smoking cessation could have a beneficial effect on lowering the incidence of dementia in the population with AF. Current guidelines substantially undervalue the role of quitting smoking in the management of AF.^19,20,21^ Therefore, we sought to determine the association between quitting smoking and the risk of incident dementia in patients with newly diagnosed AF.

## Methods

We analyzed data using the database of the Korean National Health Insurance Service.^22^ Further information regarding data sources is provided in the eMethods in the Supplement. In addition, the National Health Insurance Data Sharing Service provides access to all the data collected and analyzed in this study.^23^ This study adhered to the principles of the Declaration of Helsinki^24^ and was approved by the Seoul National University Hospital Institutional Review Board, which waived the need for informed consent because of the use of deidentified data. This study followed the Strengthening the Reporting of Observational Studies in Epidemiology (STROBE) reporting guideline.

### Study Design and Study Population

**Figure 1.**
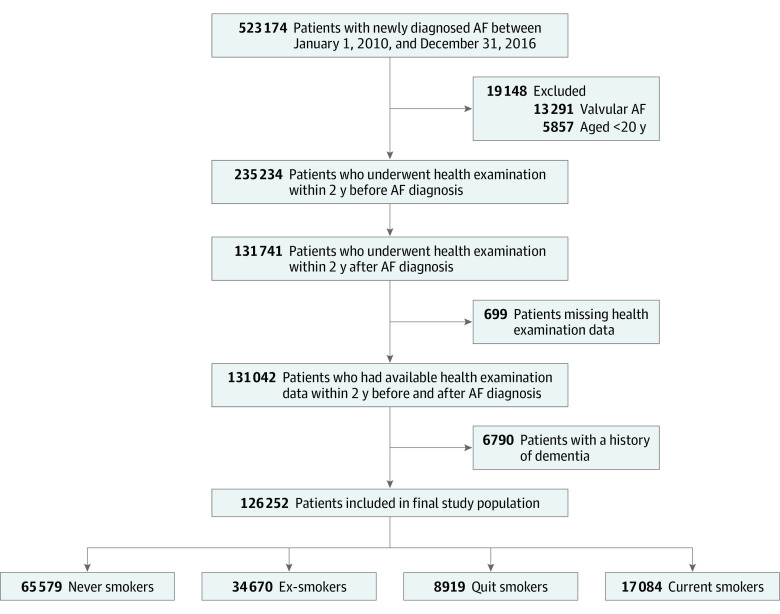
Study Enrollment Flowchart Smoking status is described in the Study Design and Study Population subsection of the Methods section. AF indicates atrial fibrillation.

The enrollment process for this study is shown in Figure 1. We identified 523 174 patients with newly diagnosed AF between January 1, 2010, and December 31, 2016. We included patients who underwent a health examination within the preceding 2 years of AF diagnosis and underwent a health examination within 2 years of AF diagnosis. However, we excluded patients with valvular AF who were younger than 20 years with missing health examination data, and with prevalent dementia. eTable 1 in the Supplement contains detailed definitions of diagnoses.^22^

**Figure 2.**
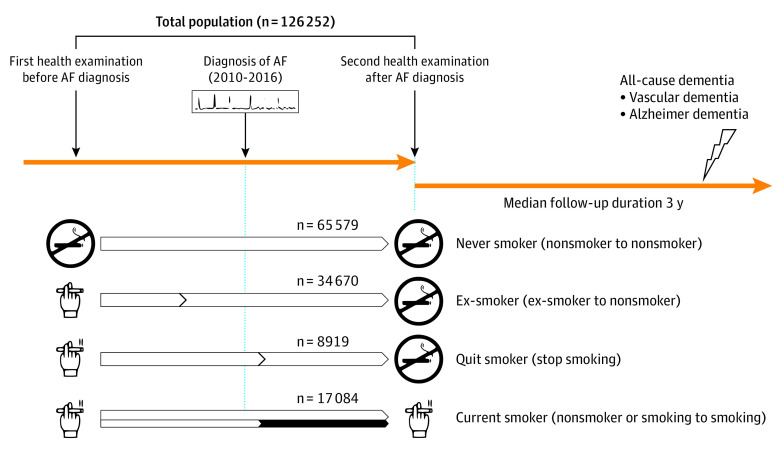
Groups Categorized by Smoking Status Before and After Atrial Fibrillation (AF) Diagnosis Smoking status is described in the Study Design and Study Population subsection of the Methods section.

Patients were categorized based on their smoking status and change before the first health examination and on AF diagnosis (second health examination) (Figure 2). Initially, the first and second health assessments used a self-reported questionnaire to assess smoking status. Then, using 2 successive questionnaires, patients were divided into 4 groups: never smokers, ex-smokers, quit smokers, and current smokers. Never smokers were defined as nonsmokers at both the initial and follow-up health assessments. Ex-smokers were defined as ex-smokers at the time of the first examination and continued to be nonsmokers at the time of the second examination. Quit smokers were those who smoked at the time of their initial health assessment but stopped after being diagnosed with AF (in the second examination). Patients who were current smokers at the time of the second assessment were deemed current smokers, regardless of their smoking status at the time of the first examination.

### Covariates

Patients’ demographic data, comorbidities, medications, and health examination results were used as covariates. The demographic characteristics, comorbidities, and medications of the patients were extracted from the Korean National Health Insurance Service database. Comorbidity definitions are summarized in eTable 1 in the Supplement.^22^ Covariates are described in detail in the eMethods in the Supplement.

### Study Outcome and Follow-up

The index date was determined to be the second health examination date. Incident dementia was identified as the primary outcome event. eTable 1 in the Supplement provides comprehensive definitions of the study outcomes.^22^ In addition, patients were tracked from the index date until dementia onset, death, or the end of the study period (December 31, 2017), whichever occurred first.

### Statistical Analysis

Data were analyzed from January 13, 2002, to March 29, 2022. Baseline characteristics are presented as the mean (SD) for continuous variables and numbers (percentages) for categorical variables. We used 1-way analysis of variance and a χ^2^ test to analyze the significant differences in smoking status among the groups. The incidence rate of dementia was determined by dividing the number of incident cases by the total follow-up period and expressed as a rate per 1000 person-years. The hazard ratios (HRs) and 95% CIs for dementia were calculated using the Cox proportional hazards regression model. Analyses were initially conducted with an unadjusted HR; then, a multivariable-adjusted proportional hazards model was applied. Model 1 was adjusted for age and sex. Model 2 was adjusted for age; sex; comorbidities, including hypertension, diabetes mellitus, dyslipidemia, heart failure, prior myocardial infarction (MI), prior stroke, peripheral artery disease, chronic kidney disease, chronic obstructive pulmonary disease, and cancer; baseline medications, including oral anticoagulants, aspirin, P2Y_12_ inhibitor, and statin; baseline body mass index (calculated as weight in kilograms divided by height in meters squared); heavy alcohol consumption; regular exercise; low income; and CHA_2_DS_2_-VASc score, which measures ischemic stroke risk in individuals with atrial fibrillation.

Hazard ratios are reported using current smokers (model 2) and never smokers (model 2a) as a reference group to demonstrate the discrepancy between each group’s relative risk and that of never smokers. We stratified each smoking group into greater than 0 to less than 10, 10 to less than 20, 20 to less than 30, and 30 or more pack-years to account for the anticipated influence of cumulative smoking amount. Because it is generally established that dementia has a preclinical phase, and the changes in smoking status might likely take time to affect the incidence of dementia,^25^ we conducted a sensitivity analysis using the landmark analysis to prevent immortal time bias. Between the index date and the landmark time point in the landmark analysis, we eliminated patients who had incident dementia, and a year was selected as the landmark time point. Time 0 was 1 year after the index date.

Statistical significance was set at 2-sided *P* < .05. The 95% CI excluding the null was considered statistically significant. For statistical analyses, we used SAS, version 9.4 (SAS Institute Inc).

### Sensitivity and Subgroup Analyses

To provide a complementary analysis, we conducted a propensity score (PS)–matching analysis to compare the risk of dementia between quit smoker and current smoker groups.^26,27^ The propensity of being in the quit smoker group was measured by a logistic regression model with all covariates included in multivariable Cox proportional hazards regression analysis. Each patient in the quit smoker group was matched to 1 patient in the current smoker group (1:1 matching). With a caliper of 0.01 of the PS, we used the greedy nearest-neighbor technique without replacement for matching.^26^ The absolute standardized difference was used to assess the balance between the 2 groups. In each covariate, an absolute standardized difference of no more than 0.1 indicates that there was no significant difference between the 2 groups.^28^ Also, for the sensitivity analysis, we used the Fine and Gray proportional hazards model for the subdistribution of a competing risk to conduct a competing risk analysis with death as a competing risk.^29^ Subgroup analyses were used to assess the possible impact modification owing to sex, age (<65 years, 65-74 years, and ≥75 years), CHA_2_DS_2_-VASc score (<3 and ≥3), and history of stroke.

## Results

We enrolled 126 252 patients in this study (mean [SD] age, 62.6 [12.0] years; 61.9% men and 38.1% women; mean [SD] CHA_2_DS_2_-VASc score, 2.7 [1.7]). Smoking status of the total study population was as follows: 65 579 never smokers (51.9%), 34 670 ex-smokers (27.5%), 8919 quit smokers (7.1%), and 17 084 current smokers (13.5%). Between the former health examination and the diagnosis of AF, the mean (SD) interval was 1.1 (0.5) years; between the diagnosis of AF and the latter health examination, 0.9 (0.5) years; and between the former and latter health examinations, 2.0 (0.5) years.

### Baseline Characteristics

Baseline characteristics are presented in the Table. Ex-smokers, quit smokers, and current smokers were younger (mean [SD] age range, 56.2 [12.5] to 63.6 [11.1] years) and had a lower CHA_2_DS_2_-VASc score (mean [SD] range, 2.0 [1.9] to 2.4 [1.6]) compared with nonsmokers (mean (SD) age, 64.4 [11.7] years; mean [SD] CHA_2_DS_2_-VASc score, 3.1 [1.7]; *P* < .001 for both). Comorbid conditions, such as hypertension (never smokers, 66.0%; ex-smokers, 67.7%; quit smokers, 68.5%; current smokers, 64.0%; *P* < .001), type 2 diabetes (never smokers, 21.6%; ex-smokers, 24.3%; quit smokers, 25.4%; current smokers, 24.0%; *P* < .001), heart failure (never smokers, 25.1%; ex-smokers, 24.8%; quit smokers, 27.5%; current smokers, 21.5%; *P* < .001), and prior MI (never smokers, 4.5%; ex-smokers, 5.4%; quit smokers, 8.6%; current smokers, 5.0%; *P* < .001), were more prevalent among quit smokers than in the other groups. Current smokers were more likely to engage in heavy alcohol consumption than the other groups (2665 [15.6%] vs 7285 [5.8%] for all). Quit smokers had the highest previous amount of smoking assessed in the first health examination (mean [SD], 24.9 [19.5] pack-years), followed by current smokers (mean [SD], 21.9 [15.7] pack-years) and ex-smokers (mean [SD], 19.9 [18.0] pack-years) (Table).

**Table.** Baseline Characteristics of the Study Population by Smoking Status

Characteristic	Smoking status^a^					*P* value
	Total (n = 126 252)	Never smoker (n = 65 579)	Ex-smoker (n = 34 670)	Quit smoker (n = 8919)	Current smoker (n = 17 084)	
Age, mean (SD), y	62.6 (12.0)	64.4 (11.7)	63.6 (11.1)	59.1 (11.9)	56.2 (12.5)	<.001
Age group, y						
<65	64 320 (50.9)	29 023 (44.3)	17 133 (49.4)	5753 (64.5)	12 411 (72.6)	<.001
65 to <75	41 259 (32.7)	23 508 (35.8)	11 827 (34.1)	2371 (26.6)	3553 (20.8)	
≥75	20 673 (16.4)	13 048 (19.9)	5710 (16.5)	795 (8.9)	1120 (6.5)	
Sex						
Men	78 191 (61.9)	19 901 (30.43	33 802 (97.5)	8354 (93.7)	16 134 (94.4)	<.001
Women	48 061 (38.1)	45 678 (69.7)	868 (2.5)	565 (6.3)	950 (5.6)	
CHA_2_DS_2_-VASc score, mean (SD)	2.7 (1.7)	3.1 (1.7)	2.4 (1.6)	2.4 (1.6)	2.0 (1.9)	<.001
CHA_2_DS_2_-VASc score						
0	8205 (6.5)	1871 (2.9)	3083 (8.9)	846 (9.5)	2405 (14.1)	<.001
1	25 508 (20.2)	10 105 (15.4)	7980 (23.0)	2084 (23.4)	5339 (31.3)	
≥2	92 539 (73.3)	53 603 (81.7)	23 607 (68.1)	5989 (67.1)	9340 (54.7)	
Comorbidities						
Hypertension	83 820 (66.4)	43 283 (66.0)	23 492 (67.7)	6106 (68.5)	10 939 (64.0)	<.001
Type 2 diabetes	28 969 (22.9)	14 175 (21.6)	8432 (24.3)	2266 (25.4)	4096 (24.0)	<.001
Dyslipidemia	13 185 (10.4)	7000 (10.7)	3141 (9.1)	984 (11.0)	2060 (12.1)	<.001
Heart failure	31 246 (24.7)	16 499 (25.1)	8612 (24.8)	2456 (27.5)	3679 (21.5)	<.001
Prior MI	6438 (5.1)	2942 (4.5)	1880 (5.4)	766 (8.6)	850 (5.0)	<.001
PAD	27 325 (21.6)	15 142 (23.1)	7227 (20.8)	1761 (19.7)	3195 (18.7)	<.001
CKD	18 828 (14.9)	11 268 (17.2)	4831 (13.9)	1186 (13.3)	1543 (9.0)	<.001
COPD	24 542 (19.4)	12 605 (19.2)	6888 (19.9)	2059 (23.1)	2990 (17.5)	<.001
Cancer	7235 (5.7)	3694 (5.6)	2299 (6.6)	729 (8.2)	513 (3.0)	<.001
Medication						
OAC	35 411 (28.01)	17 967 (27.4)	10 673 (30.8)	3000 (33.6)	3771 (22.1)	<.001
Warfarin	26 109 (20.7)	12 835 (19.6)	7864 (22.7)	2422 (27.2)	2988 (17.5)	<.001
DOAC	12 297 (9.7)	6718 (10.2)	3730 (10.7)	822 (9.2)	1027 (6.0)	<.001
Aspirin	26 679 (21.1)	13 450 (20.5)	7827 (22.6)	1889 (21.2)	3513 (20.6)	<.001
P2Y_12_ inhibitor	8842 (7.0)	4303 (6.6)	2622 (7.6)	838 (9.4)	1079 (6.3)	<.001
Statin	23 492 (18.6)	12 687 (19.3)	6326 (18.2)	1789 (20.1)	2690 (15.7)	<.001
Health examination parameter, mean (SD)						
BMI	24.5 (3.3)	24.52 (3.4)	24.65 (3.1)	24.62 (3.4)	24.4 (3.4)	<.001
Waist circumference, cm	84.5 (9.3)	82.86 (9.8)	86.68 (8.2)	86.36 (8.7)	85.7 (8.7)	<.001
Blood pressure, mm Hg						
Systolic	125.7 (15.5)	126.1 (16.0)	126.1 (14.8)	124.7 (15.2)	124.14 (14.7)	<.001
Diastolic	77.1 (10.3)	76.8 (10.3)	77.4 (10.2)	77.2 (10.3)	77.4 (10.3)	<.001
Fasting glucose level, mg/dL	105.0 (27.3)	103.7 (26.4)	105.9 (25.8)	106.5 (29.0)	107.11 (32.2)	<.001
Total cholesterol level, mg/dL	181.0 (40.7)	183.6 (40.5)	176.2 (40.1)	178.6 (41.9)	182.2 (41.4)	<.001
LDL-C level, mg/dL	103.2 (41.2)	105.6 (40.9)	100.1 (40.7)	100.2 (38.1)	101.8 (43.9)	<.001
HDL-C level, mg/dL	52.2 (15.0)	53.6 (15.0)	50.9 (15.4)	50.0 (13.0)	50.3 (14.8)	<.001
eGFR, mL/min/1.73 m^2^	80.3 (28.1)	79.4 (28.0)	79.4 (27.4)	81.6 (31.4)	85.0 (27.3)	<.001
Smoking amount, mean (SD), pack-years	10.2 (16.2)	0 (0)	19.9 (18.0)	24.9 (19.5)	21.9 (15.7)	<.001
Smoking amount, pack-years						
0	65 579 (51.9)	65 579 (100)	0	0	0	<.001
>0 to <10	15 550 (12.3)	0	10 178 (29.4)	1795 (20.1)	3577 (20.9)	<.001
10 to <20	15 555 (12.3)	0	8870 (25.6)	2038 (22.9)	4647 (27.2)	<.001
20 to <30	11 738 (9.3)	0	6404 (18.5)	1689 (18.9)	3645 (21.3)	<.001
≥30	17 830 (14.1)	0	9218 (26.6)	3397 (38.1)	5215 (30.5)	<.001
Alcohol consumption						<.001
None	84 232 (66.7)	54 739 (83.5)	18 344 (52.9)	5025 (56.3)	6124 (35.8)	<.001
Mild to moderate	34 735 (27.5)	9793 (14.9)	13 475 (38.9)	3172 (35.6)	8295 (48.5)	<.001
Heavy	7285 (5.8)	1047 (1.6)	2851 (8.2)	722 (8.1)	2665 (15.6)	<.001
Regular exercise	21 223 (16.8)	12 729 (19.4)	9867 (28.5)	2056 (23.1)	3376 (19.8)	<.001
Low income	21 223 (16.8)	11 422 (17.4)	5244 (15.1)	1500 (16.8)	3057 (17.9)	<.001

Abbreviations: BMI, body mass index (calculated as weight in kilograms divided by height in meters squared); CKD, chronic kidney disease; COPD, chronic obstructive pulmonary disease; DOAC, direct oral anticoagulant; eGFR, estimated glomerular filtration rate; HDL-C, high-density lipoprotein cholesterol; LDL-C, low-density lipoprotein cholesterol; MI, myocardial infarction; OAC, oral anticoagulant; PAD, peripheral artery disease.

SI conversion factors: To convert cholesterol levels to mmol/L, multiply by 0.0259; glucose to mmol/L, multiply by 0.0555.

^a^ Data are expressed as No. (%) of participants unless indicated otherwise. Percentages have been rounded and may not total 100.

### Association Between Smoking Status Changes and Risk of Incident Dementia

**Figure 3.**
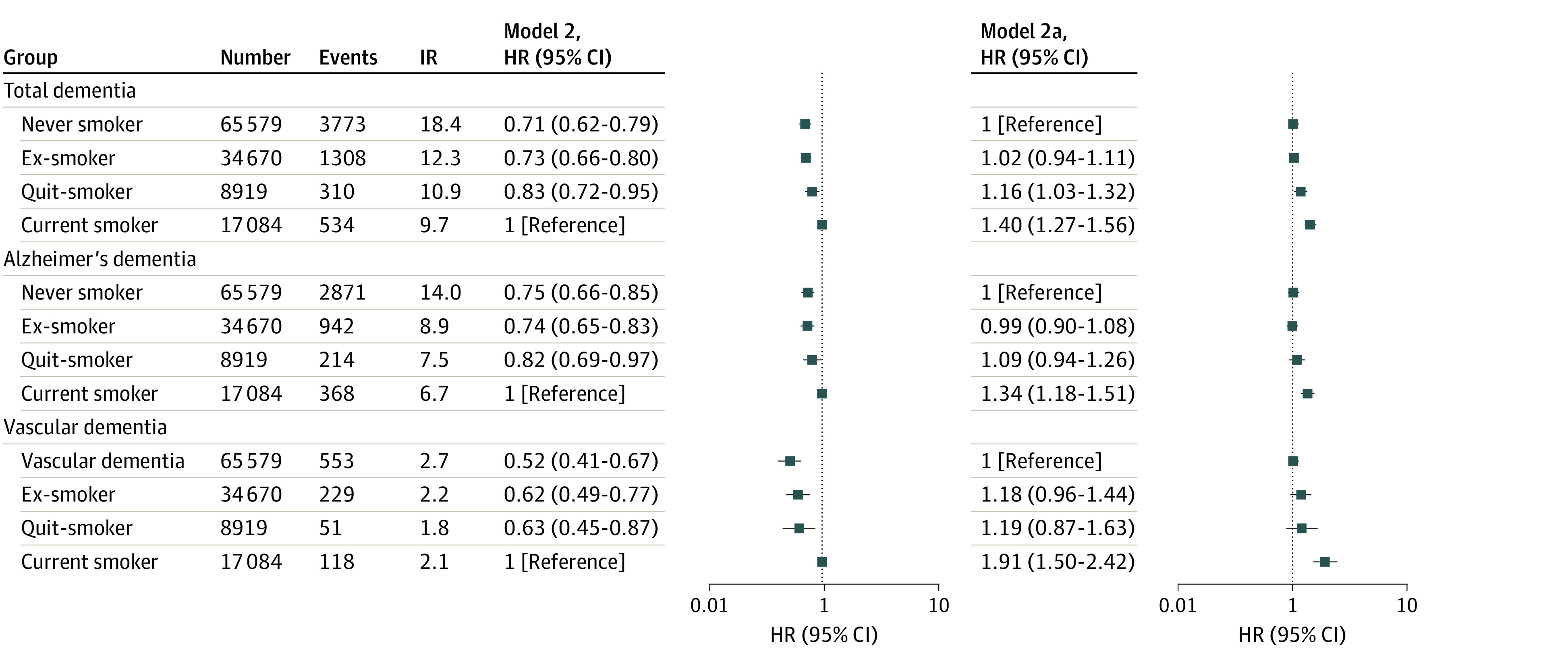
Hazard Ratios (HRs) of Smoking Status on the Risk of Total Dementia, Alzheimer Disease, and Vascular Dementia Incidence rate (IR) is calculated per 1000 person-years. Smoking status is described in the Study Design and Study Population subsection of the Methods section. In model 2a, never smokers constituted the reference group.

Dementia occurred in 5925 patients during a mean (SD) follow-up period of 3.1 (1.9) years (incidence rate, 1.11 per 1000 person-years). There were 4395 patients with Alzheimer disease and 951 with vascular dementia among the 5925 patients (incidence rates, 1.5 and 0.24 per 1000 person-years, respectively). eTable 2 in the Supplement and Figure 3 present crude event numbers, incidence rates, and HRs for total dementia, Alzheimer disease, and vascular dementia according to smoking status.

After multivariable adjustment, current smokers were shown to have the highest risk of dementia compared with never smokers (HR, 1.41 [95% CI, 1.27-1.56]) (Figure 3). Quit smokers also showed a higher risk of dementia (HR, 1.16 [95% CI, 1.03-1.32]) than never smokers. Ex-smokers did not have a statistically significantly higher risk than never smokers (HR, 1.02 [95% CI, 0.94-1.11]). Consequently, quit smokers had a substantially reduced risk of dementia (by 17%) compared with current smokers (HR, 0.83 [95% CI, 0.72-0.95]).

Although the association of smoking status was consistently observed with the risk of Alzheimer disease and vascular dementia, the positive association of smoking cessation was more accentuated for the 38% reduced risk of vascular dementia (HR, 0.63 [95% CI, 0.45-0.87]) than for Alzheimer disease (19% reduced risk) (HR, 0.81 [95% CI, 0.69-0.97]). The results of the sensitivity analysis with the 1-year landmark analysis were consistent with the main results (eFigure 1 and eTable 4 in the Supplement).

### Sensitivity Analysis

After PS matching, baseline characteristics between the quit smoker and current smoker groups were well balanced. The absolute standardized differences for all covariates were less than 0.01; thus, the differences were negligible between the 2 groups in all covariates (eTable 5 in the Supplement). For total dementia, Alzheimer disease, and vascular dementia, the current smoker group showed higher incidence rates than the quit smoker group (12.8 vs 10.9 per 1000 person years) (eTable 6 in the Supplement). Compared with current smoking, quitting smoking was associated with a 15% lower risk of total dementia (HR, 0.85 [95% CI, 0.73-0.99]) (eTable 6 in the Supplement). In terms of subtype of dementia, quitting smoking was associated with a significantly lower risk of vascular dementia by 36% compared with current smokers (HR, 0.64 [95% CI, 0.45-0.91]). Quit smokers tended to have a lower risk of Alzheimer disease than current smokers, but this was not statistically significant (HR, 0.84 [95% CI, 0.69-1.01]). The results of the PS matching analyses were largely consistent with the main analysis by the multivariable Cox proportional hazards regression analysis. When competing risks of death were taken into account, the results remained consistent (eTable 7 in the Supplement).

### Subgroup Analysis

eFigures 2 to 4 in the Supplement present the results of subgroup analyses. Although the number of patients was small in specific subgroups and the 95% CIs were wider than the main results, subgroup analyses largely corroborated the main results (eFigure 2 in the Supplement). Current smoking was consistently associated with the highest risk of dementia compared with never smoking across the various subgroups. Conversely, quitting smoking consistently tended to be associated with a lower incidence of dementia than current smoking in various subgroups.

### Smoking Amount Assessed at First Health Examination and Dementia Risk

**Figure 4.**
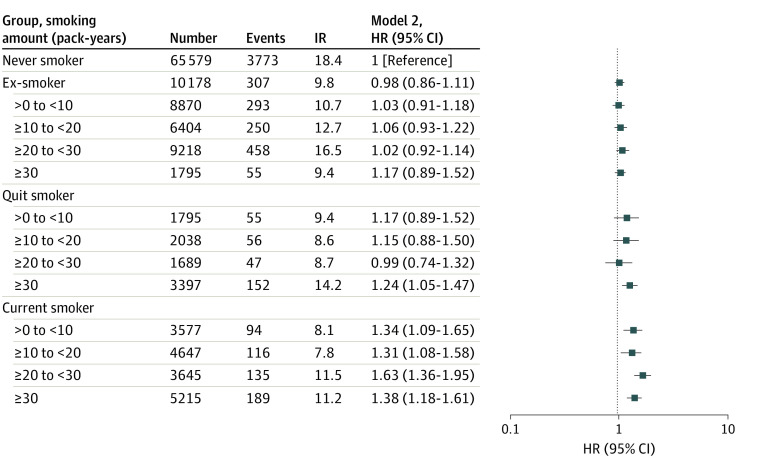
Hazard Ratios (HRs) of Smoking Status on the Risk of Total Dementia According to Pack-Years Smoked Incidence rate (IR) is calculated per 1000 person-years. Smoking status is described in the Study Design and Study Population subsection of the Methods section.

For each group of smokers (ie, ex-smokers, quit smokers, and current smokers), smoking amount was stratified by pack-years smoked (>0 to <10, 10 to <20, 20 to <30, and ≥30) and assessed at the first health examination. Being an ex-smoker with any previous smoking amount was not associated with a significantly higher incidence of dementia compared with being a never smoker (Figure 4 and eTable 3 in the Supplement). Current smoking with any previous smoking amount was still associated with a significantly higher incidence of dementia compared with never smoking (eg, HR for ≥30 pack-years, 0.61 [95% CI, 0.52-0.70]).

Among smokers, a previous smoking amount of 30 or more pack-years was associated with a significantly higher risk of dementia than never smoking (HR, 1.24 [95% CI, 1.05-1.47]). On the contrary, among quit smokers, a previous smoking amount of less than 30 pack-years was not associated with a substantially higher incidence of dementia than never smoking (eg, HR for 20 to <30 pack-years, 0.99 [95% CI, 0.74-1.32]).

## Discussion

To the best of our knowledge, this is the first and largest study to demonstrate the clinical significance of smoking status and its changes after AF diagnosis on the incidence of dementia. The principal findings of our study are as follows: on being diagnosed with AF, a considerable proportion of patients (13.5%) continued to smoke, whereas 7.1% of patients quit smoking; current smoking was associated with the highest risk of all-cause dementia, including Alzheimer disease and vascular dementia, than all other smoking status groups; quitting smoking after AF diagnosis was associated with a substantially reduced risk of all-cause dementia (by 17%), Alzheimer disease (by 19%), and vascular dementia (by 38%); and the beneficial association of quitting smoking after AF diagnosis with reduced risk of incident dementia was consistently observed in various subgroups when stratified by sex, age, CHA_2_DS_2_-VASc score, and history of stroke.

Several baseline covariates showed differences among the different smoking status groups. For example, heavy alcohol consumption was more common and lack of physical activity was less common in the current smoker group. Also, the current smoker group was younger than the quit smoker group. The rates of MI and peripheral artery disease were lower among current smokers than quit smokers. Smoking is a well-known risk factor for incident MI and peripheral artery disease. However, the mean age was higher in the quit smoker group, as was the prevalence of hypertension and diabetes, which are also well-known risk factors for MI and peripheral artery disease. We additionally performed PS matching analysis for the comparison between quit smokers and current smokers. After PS matching including age, MI, peripheral artery disease, and other variables, the findings were also consistent with the results of the main analysis.

Several previous studies have shown an elevated incidence of dementia in the population with AF.^7,8,9^ The risk of dementia increases after stroke, which has also been associated with AF.^30^ Smoking is well known to increase the risk of incident AF, stroke, and dementia.^16,17,18^ The relative risk of smoking on the risk of AF ranges from 1.3 to 1.5.^16,18^ Further, compared with never smokers, current smokers had a 30% heightened risk of all-cause dementia.^17^ Considering the close association between smoking and AF, stroke, and dementia, it would be plausible that smoking cessation may benefit patients with AF by lowering their risk of dementia. Given the many populations with AF and its increasing prevalence, efforts to prevent dementia are critical for mitigating the long-term socioeconomic and health care burden associated with AF.

The initial period after a new AF diagnosis is made crucial for the implementation of immediate, optimal care. Within 6 months of AF diagnosis, the HR for ischemic stroke was 13.28 (95% CI, 10.89-16.20), declining to 3.31 (95% CI, 3.23-3.39) after 6 months, compared with patients without AF in each period.^9^ Cardiovascular and comorbidity optimization, including lifestyle changes, have been highlighted in recent AF guidelines for holistic management.^20^ From a patient-oriented approach, on being diagnosed with a cardiac condition such as AF, patients would often move toward a healthy lifestyle. Thus, lifestyle modification in the early phase, if proactively recommended by physicians, would be a critical motivation for such patients.

Although recent studies have reported the clinical significance of lifestyle modifications in individuals with AF,^31,32,33,34,35^ current recommendations seem to disregard the effect of behavior change. For example, although smoking is listed as a risk factor for stroke in individuals with AF in current guidelines, there are no firm recommendations for smoking. However, lifestyle changes, such as restricting consumption of alcohol, managing blood pressure, and weight loss, were broadly emphasized.^20^ Additional data to improve evidence-based guideline recommendations are needed to encourage patients newly diagnosed with AF to quit smoking. The present study supports smoking cessation as a “must-have item” after an AF diagnosis to reduce the burden of AF-related dementia.

Previous studies focusing on the association of smoking and dementia have largely been performed among generally healthy populations and have demonstrated a decrease in the prevalence and risk of dementia after smoking cessation.^36,37^ In a Japanese study,^36^ current smokers were shown to have a greater risk of dementia when compared with never smokers (HR, 1.46 [95% CI, 1.17-1.80]). Among ex-smokers, the risk for those who had stopped smoking for 2 years or less remained high (HR, 1.39 [95% CI, 0.96-2.01]); however, quitting smoking for 3 years or longer mitigated the increased risk of smoking.^36^ Several studies have also demonstrated the influence of smoking status on cardiovascular outcomes in the population with AF.^32,38^ Smoking doubled the incidence of thromboembolic events in individuals with anticoagulated AF in 1 clinical trial cohort.^38^ Quit smokers also had a reduced risk of fatal ischemic stroke and cerebrovascular mortality than current smokers.^35^

We explored the extent to which AF per se compounds the risk of dementia among current smokers. Cardioembolic stroke and microembolism are the primary causes of poststroke dementia and vascular dementia in patients with AF. Approximately 45% of cardiogenic strokes are due to AF.^39^ Silent cerebral ischemia on magnetic resonance imaging is closely associated with cognitive decline in patients with AF.^40,41,42^ Several circulating biomarkers of oxidative stress, inflammation, and endothelial dysfunction are elevated during AF, which have been suggested to be associated with Alzheimer disease in previous studies.^43^ Therefore, AF may provide a specific environment for non–stroke-related cognitive decline and dementia. In our study, smoking cessation was associated with a more prominent lower risk of vascular dementia than Alzheimer disease, suggesting that smoking contributes more to the development of vascular dementia.

### Strengths and Limitations

This study has several strengths. First, we included a large sample population. Second, we confirmed the consistency of the main results in various subgroups and evaluated the dose-response association between the cumulative smoking amount and the risk of dementia in each smoking status group on a large scale. Third, given the ethical and logistic issues of conducting a nationwide clinical intervention study for lifestyle modification, our findings reemphasize the risk for dementia among patients who have AF and also smoke. Nevertheless, it also suggests an impact of smoking cessation on incident dementia in patients with AF. These findings support the increasing move toward holistic care for AF, stroke, and other cardiovascular diseases.^44,45^

This study also has several limitations. First, because this was a retrospective cohort study, the association between smoking cessation and decreased risk of dementia is based on associations rather than causation. Given the ethical implications of lifestyle modification research, we could not prove causation (eg, by randomizing patients with AF to proactively continue smoking). Second, residual confounding factors may have existed; however, we used a stepwise approach to progressively apply clinical variables to the given variables, and the main findings were consistent across several multivariable-adjusted models. Finally, although prescription of oral anticoagulants was suboptimal, this has been consistently observed in previous reports from Asian clinical practice.^46,47,48^

## Conclusions

The findings of this cohort study suggest that all types of smoking status were associated with a significantly higher risk of dementia in patients with new-onset AF. However, smoking cessation after AF diagnosis was associated with a lower risk of dementia than current smoking. These findings may support the promotion of smoking cessation to lower the risk of dementia in patients with newly diagnosed AF.

## Notes

Supplement. **eMethods.** Data Source and Covariates **eTable 1.** Definitions of Covariates **eTable 2.** Hazard Ratios of Dementia: Unadjusted and Models 1 and 2 **eTable 3.** Hazard Ratios of Dementia in Each Group Stratifying by the Smoking Amount **eTable 4.** Sensitivity Analysis With 1-Year Landmark Analysis: Hazard Ratios of Dementia at Landmark Time: Unadjusted and Models 1 and 2 **eTable 5.** Baseline Characteristics of Quit and Current Smoker Groups Before and After Propensity Score Matching **eTable 6.** Event Number, Incidence Rate, and Hazard Ratio of Dementia After Propensity Score Matching **eTable 7.** Competing Risk Analysis **eFigure 1.** Sensitivity Analysis With 1-Year Landmark Analysis: Hazard Ratios of Smoking Status on the Risk of Total Dementia, Alzheimer Disease, and Vascular Dementia **eFigure 2.** Subgroup Analyses by Sex, Age, CHA_2_DS_2_-VASc Score, and Previous Stroke (Total Dementia) **eFigure 3.** Subgroup Analyses by Sex, Age, CHA_2_DS_2_-VASc Score, and Previous Stroke (Alzheimer Disease) **eFigure 4.** Subgroup Analyses by Sex, Age, CHA_2_DS_2_-VASc Score, and Previous Stroke (Vascular Dementia)
eReferences
