# Supplementary material for: Risk of Dementia After Smoking Cessation in Patients With Newly Diagnosed Atrial Fibrillation
Source: JAMA Netw Open. 2022 Jun 15;5(6):e2217132. doi: 10.1001/jamanetworkopen.2022.17132 (PMC9201679; doi:10.1001/jamanetworkopen.2022.17132)
Supplement: Supplement. — eMethods. Data Source and Covariates eTable 1. Definitions of Covariates eTable 2. Hazard Ratios of Dementia: Unadjusted and Models 1 and 2 eTable 3. Hazard Ratios of Dementia in Each Group Stratifying by the Smoking Amount eTable 4. Sensitivity Analysis With 1-Year Landmark Analysis: Hazard Ratios of Dementia at Landmark Time: Unadjusted and Models 1 and 2 eTable 5. Baseline Characteristics of Quit and Current Smoker Groups Before and After Propensity Score Matching eTable 6. Event Number, Incidence Rate, and Hazard Ratio of Dementia After Propensity Score Matching eTable 7. Competing Risk Analysis eFigure 1. Sensitivity Analysis With 1-Year Landmark Analysis: Hazard Ratios of Smoking Status on the Risk of Total Dementia, Alzheimer Disease, and Vascular Dementia eFigure 2. Subgroup Analyses by Sex, Age, CHA2DS2-VASc Score, and Previous Stroke (Total Dementia) eFigure 3. Subgroup Analyses by Sex, Age, CHA2DS2-VASc Score, and Previous Stroke (Alzheimer Disease) eFigure 4. Subgroup Analyses by Sex, Age, CHA2DS2-VASc Score, and Previous Stroke (Vascular Dementia) eReferences [file jamanetwopen-e2217132-s001.pdf]

Supplementary Online Content

Lee HJ, Lee SR, Choi EK, et al. Risk of dementia after smoking cessation in patients with newly diagnosed atrial fibrillation. *JAMA Netw Open*. 2022;5(6):e2217132. doi:10.1001/jamanetworkopen.2022.17132

eMethods. Data Source and Covariates
eTable 1. Definitions of Covariates
eTable 2. Hazard Ratios of Dementia: Unadjusted and Models 1 and 2
eTable 3. Hazard Ratios of Dementia in Each Group Stratifying by the Smoking Amount
eTable 4. Sensitivity Analysis With 1-Year Landmark Analysis: Hazard Ratios of Dementia at Landmark Time: Unadjusted and Models 1 and 2
eTable 5. Baseline Characteristics of Quit and Current Smoker Groups Before and After Propensity Score Matching
eTable 6. Event Number, Incidence Rate, and Hazard Ratio of Dementia After Propensity Score Matching
eTable 7. Competing Risk Analysis
eFigure 1. Sensitivity Analysis With 1-Year Landmark Analysis: Hazard Ratios of Smoking Status on the Risk of Total Dementia, Alzheimer Disease, and Vascular Dementia
eFigure 2. Subgroup Analyses by Sex, Age, CHA_2DS_2-VASc Score, and Previous Stroke (Total Dementia)
eFigure 3. Subgroup Analyses by Sex, Age, CHA_2DS_2-VASc Score, and Previous Stroke (Alzheimer Disease)
eFigure 4. Subgroup Analyses by Sex, Age, CHA_2DS_2-VASc Score, and Previous Stroke (Vascular Dementia)
eReferences

This supplementary material has been provided by the authors to give readers additional information about their work.

© 2022 Lee HJ et al. *JAMA Network Open*.

eMethods. Data Source and Covariates

Data Source

In brief, the government provides mandatory health insurance coverage to the entire Korean population, approximately 50 million people. The Korean NHIS database contains demographic data as well as information about enrollees' medical expenses, such as diagnoses, examinations, prescription dispensing records, and inpatient and outpatient procedure records.[1,2] The International Classification of Diseases, Tenth Revision, Clinical Modification codes were used to classify diagnoses. Additionally, enrollees aged 40 years or older are recommended to undergo a biannual health screening conducted by the Korean National Health Insurance Corporation. The health examination consists of anthropometric measurements, blood pressure, a laboratory examination, and a chest X-ray, as well as a self-reported questionnaire on health behaviors such as smoking, alcohol consumption, and physical activity. In 2014, 75% of participants participated.[2] These data are linked with the Korean NHIS database as a National Health Screening database.

Covariates

Comorbidities included hypertension, diabetes mellitus, dyslipidemia, heart failure, prior myocardial infarction (MI), peripheral artery disease (PAD), chronic kidney disease (CKD), chronic obstructive pulmonary disease (COPD), and cancer.[3-6] The CHA_2DS_2-VASc score for assessing stroke risk was calculated combining covariate information (**Online Table 1**).[7] Baseline medications, including OACs (i.e. warfarin or direct oral anticoagulants, DOACs), aspirin, $P2Y_{12}$ inhibitors, and statin, were identified between the date of AF diagnosis and the date of the second health examination. As patients' baseline variables, body mass index, waist circumference, systolic and diastolic blood pressure, fasting glucose, total cholesterol, low-density lipoprotein cholesterol, high-density lipoprotein cholesterol, and estimated glomerular filtration rate at the second health examination were collected. Alcohol consumption was categorized into non-drinker, mild to moderate drinker (>0g/day to <30 g/day), and heavy drinker (≥30 g/day). Regular exercise was defined as performing >30 minutes of moderate physical activity ≥5 times a week or >20 minutes of strenuous physical activity ≥5 times per week. Low income was defined as having income in lower 20% among the entire Korean population of subjects supported by the Medical Aid program.

© 2022 Lee HJ et al. *JAMA Network Open*.

eTable 1. Definitions of Covariates

Diagnosis	*ICD-10-CM* code and definition	Diagnostic definition
**Inclusion/exclusion criteria**		
**Atrial fibrillation**	I48.0-48.4, I48.9	Admission≥1 or outpatient department≥2
**Valvular atrial fibrillation**	I05.0, I05.2, I05.9, Z95.2-Z95.4	Admission or outpatient department≥1
**Dementia**	F00-03, F107, G30, G231, G310, G311, G318 and prescription of medication for dementia, including galantamine, rivastigmine, donepezil, or memantine	Admission or outpatient department ≥1
**Comorbidities**		
**Hypertension**	I10-I13, I15; and minimum 1 prescription of anti-hypertensive drug (thiazide, loop diuretics, aldosterone antagonist, alpha-/beta-blocker, calcium-channel blocker, angiotensin-converting enzyme inhibitor, angiotensin II receptor blocker).	Admission≥1 or outpatient department≥2
	or systolic/diastolic blood pressure ≥ 140/90 mmHg	Based on the results of 2nd health exam
**Diabetes mellitus**	E11-E14; and minimum 1 prescription of anti-diabetic drugs (sulfonylureas, metformin, meglitinides, thiazolidinediones, dipeptidyl peptidase-4 inhibitors, α-glucosidase inhibitors, and insulin).	Admission≥1 or outpatient department≥2

© 2022 Lee HJ et al. *JAMA Network Open*.

	or fasting glucose level ≥ 126 mg/dL	Based on the results of 2nd health exam
**Dyslipidemia**	E78	Admission or outpatient department≥1
	or Total cholesterol ≥ 240 mg/dL	Based on the results of 2nd health exam
**Heart failure**	I50	Admission or outpatient department≥1
**Vascular disease**		
**Prior MI**	I21, I22	Admission or outpatient department≥1
**PAD**	I70, I73	Admission or outpatient department≥2
**CKD**	eGFR<60ml/min/1.73m^2	Based on the results of 2nd health exam
**COPD**	J41-44	Admission≥1
**Cancer**	C00-97 and RID code (V193)	Admission or outpatient department≥1
**Health exam questionnaire**		
**Alcohol consumption**	Mild to moderate drinker: alcohol consumption >0g to <30g per day	Based on the results of 2nd health exam
	Heavy drinker: alcohol consumption ≥30g per day	

© 2022 Lee HJ et al. *JAMA Network Open*.

**Regular exercise**	Performing a moderate physical activity more than 30 minutes at least 5 times per week or strenuous physical activity more than 20 minutes at least 3 times per week.	Based on the results of 2nd health exam
**Low income**	Income lowest 20% and medical aid	
**Scores**		
**CHA_2DS_2-VASc score**	Heart failure (1 point), hypertension (1 point), age ≥75 years (2 points), diabetes (1 point), previous stroke/systemic embolism/transient ischemic attack (2 points), vascular disease (prior MI or PAD, 1 point) and female sex (1 point)	
**Clinical outcome**		
**Dementia**	F00-03, F107, G30, G231, G310, G311, G318 and prescription of medication for dementia, including galantamine, rivastigmine, donepezil, or memantine	Admission or outpatient department ≥1
**Alzheimer Disease**	F00, G30 and prescription of medication for dementia, including galantamine, rivastigmine, donepezil, or memantine	Admission or outpatient department ≥1
**Vascular Dementia**	F01 and prescription of medication for dementia, including galantamine, rivastigmine, donepezil, or memantine	Admission or outpatient department ≥1

Abbreviation: CKD, chronic kidney disease; COPD, chronic obstructive pulmonary disease; CT, computed tomography; eGFR, estimated glomerular filtration rate; MI, myocardial infarction; MRI, magnetic resonance image; PAD, peripheral artery disease.

© 2022 Lee HJ et al. *JAMA Network Open*.

eTable 2. Hazard Ratios of Dementia: Unadjusted and Models 1 and 2

Smoking status	Number	Events	IR	Unadjusted HR (95% CI)	Model 1 HR (95% CI)	Model 2 HR (95% CI)
**Dementia**				P=<0.001	P=<0.001	P=<0.001
**Never smoker**	65579	3773	18.4	1.898 (1.734,2.078)	0.673 (0.607,0.746)	1 (reference)
**Ex-smoker**	34670	1308	12.3	1.277 (1.155,1.412)	0.689 (0.622,0.762)	1.019 (0.940,1.106)
**Quitter**	8919	310	10.9	1.128 (0.980,1.297)	0.866 (0.753,0.996)	1.163 (1.026,1.317)
**Current smoker**	17084	534	9.7	1 (reference)	1 (reference)	1.406 (1.267,1.560)
**A-Dementia**				P=<0.001	P=<0.001	P=<0.001
**Never smoker**	65579	2871	14	2.097 (1.881,2.337)	0.707 (0.626,0.799)	1 (reference)
**Ex-smoker**	34670	942	8.9	1.336 (1.184,1.507)	0.700 (0.620,0.790)	0.986 (0.896,1.084)
**Quitter**	8919	214	7.5	1.130 (0.955,1.338)	0.855 (0.722,1.012)	1.089 (0.939,1.263)
**Current smoker**	17084	368	6.7	1 (reference)	1 (reference)	1.336 (1.181,1.512)
**V-Dementia**				P=<0.001	P=<0.001	P=<0.001
**Never smoker**	65579	553	2.7	1.258 (1.032,1.535)	0.512 (0.405,0.649)	1 (reference)
**Ex-smoker**	34670	229	2.2	1.011 (0.809,1.262)	0.603 (0.482,0.755)	1.177 (0.961,1.442)
**Quitter**	8919	51	1.8	0.840 (0.605,1.166)	0.674 (0.485,0.936)	1.193 (0.874,1.629)
**Current smoker**	17084	118	2.1	1 (reference)	1 (reference)	1.908 (1.503,2.423)

Model 1: age and sex-adjusted

Model 2: age, sex, body mass index, heavy drinker, regular physical activity, low income, diabetes, hypertension, dyslipidemia, previous myocardial infarction, previous stroke, previous congestive heart failure, peripheral artery disease, chronic obstructive pulmonary disease, cancer, chronic kidney disease, CHA2DS2-VASc score, warfarin, non–vitamin K antagonist oral anticoagulants, aspirin, clopidogrel, statin.

Abbreviation: CI, confidence interval; HR, hazard ratio; IR, incidence rate.

© 2022 Lee HJ et al. *JAMA Network Open*.

eTable 3. Hazard Ratios of Dementia in Each Group Stratifying by the Smoking Amount

Smoking status	Smoking amount (pack-years)	Number	Events	IR	Unadjusted HR (95% CI)	Model 1 HR (95% CI)	Model 2 HR (95% CI)
**Dementia**					<0.001	<0.001	<0.001
**Never smoker**	**0**	65579	3773	18.4	1 (reference)	1 (reference)	1 (reference)
**Ex-smoker**	**>0 to <10**	10178	307	9.8	0.532 (0.474,0.598)	0.958 (0.845,1.087)	0.978 (0.862,1.109)
	**≥10 to <20**	8870	293	10.7	0.586 (0.520,0.660)	1.026 (0.901,1.169)	1.032 (0.907,1.175)
	**≥20 to <30**	6404	250	12.7	0.694 (0.611,0.789)	1.06 (0.923,1.218)	1.064 (0.927,1.223)
	**≥30**	9218	458	16.5	0.904 (0.820,0.996)	1.061 (0.949,1.185)	1.024 (0.916,1.145)
**Quitter**	**>0 to <10**	1795	55	9.4	0.509 (0.39,0.664)	1.252 (0.958,1.636)	1.165 (0.891,1.522)
	**≥10 to <20**	2038	56	8.6	0.469 (0.361,0.611)	1.288 (0.986,1.683)	1.148 (0.879,1.501)
	**≥20 to <30**	1689	47	8.7	0.476 (0.357,0.634)	1.083 (0.810,1.450)	0.988 (0.738,1.322)
	**≥30**	3397	152	14.2	0.777 (0.661,0.914)	1.389 (1.171,1.648)	1.242 (1.046,1.474)
**Current smoker**	**>0 to <10**	3577	94	8.1	0.439 (0.357,0.538)	1.434 (1.164,1.766)	1.338 (1.086,1.648)
	**≥10 to <20**	4647	116	7.8	0.425 (0.353,0.511)	1.365 (1.128,1.652)	1.307 (1.079,1.583)
	**≥20 to <30**	3645	135	11.5	0.628 (0.529,0.745)	1.736 (1.451,2.077)	1.633 (1.364,1.954)
	**≥30**	5215	189	11.2	0.607 (0.524,0.702)	1.454 (1.244,1.699)	1.379 (1.178,1.614)

IR, per 1000 person-years

Model 1: age and sex-adjusted

Model 2: age, sex, hypertension, diabetes mellitus, dyslipidemia, heart failure, prior MI, PAD, CKD, COPD, cancer, BMI, heavy drinker, regular exercise, low income, use of OAC, use of antiplatelet agent, use of statin, and CHA_2DS_2-VASc score.

Abbreviation: CI, confidence interval; HR, hazard ratio; IR, incidence rate.

© 2022 Lee HJ et al. *JAMA Network Open*.

Table 4. Sensitivity Analysis With 1-Year Landmark Analysis: Hazard Ratios of Dementia at Landmark Time: Unadjusted and Models 1 and 2

Smoking status	Unadjusted HR (95% CI)	Model 1 HR (95% CI)	Model 2 HR (95% CI)
**Dementia**			
**Never smoker**	1.872 (1.687,2.078)	0.666 (0.591,0.750)	0.697 (0.618,0.785)
**Ex-smoker**	1.297 (1.156,1.456)	0.700 (0.623,0.787)	0.733 (0.652,0.824)
**Quit-smokers**	1.105 (0.940,1.299)	0.843 (0.717,0.991)	0.805 (0.684,0.947)
**Current smoker**	1 (reference)	1 (reference)	1 (reference)
**Alzheimer disease**			
**Never smoker**	2.082 (1.837,2.359)	0.715 (0.621,0.824)	0.752 (0.652,0.866)
**Ex-smoker**	1.388 (1.209,1.594)	0.728 (0.634,0.837)	0.765 (0.665,0.880)
**Quit-smokers**	1.140 (0.939,1.383)	0.857 (0.706,1.040)	0.814 (0.670,0.988)
**Current smoker**	1 (reference)	1 (reference)	1 (reference)
**Vascular dementia**			
**Never smoker**	1.274 (1.014,1.602)	0.492 (0.374,0.647)	0.492 (0.373,0.648)
**Ex-smoker**	1.033 (0.800,1.334)	0.614 (0.475,0.795)	0.618 (0.476,0.802)
**Quit-smokers**	0.746 (0.502,1.108)	0.593 (0.399,0.881)	0.552 (0.371,0.821)
**Current smoker**	1 (reference)	1 (reference)	1 (reference)

Model 1: age and sex-adjusted

Model 2: age, sex, hypertension, diabetes mellitus, dyslipidemia, heart failure, prior MI, PAD, CKD, COPD, cancer, BMI, heavy drinker, regular exercise, low income, use of OAC, use of antiplatelet agent, use of statin, and CHA_2DS_2-VASc score.

Abbreviation: CI, confidence interval; HR, hazard ratio

All, p for trends < 0.001

© 2022 Lee HJ et al. *JAMA Network Open*.

eTable 5. Baseline Characteristics of Quit and Current Smoker Groups Before and After Propensity Score Matching

	**Before PS matching**			**After PS matching**		
	**Quit-smoker**	**Current smoker**	**ASD**	**Quit-smoker**	**Current smoker**	**ASD**
**Number**	8918	17084		8573	8573	
**Age (years)**	59.1 (11.9)	56.2 (12.5)	0.234	58.8 (11.9)	58.9 (12.1)	0.009
**<65 years**	5753 (64.5)	12,411 (72.7)		5606 (65.4)	5560 (64.9)	
**65 to <75 years**	2371 (26.6)	3553 (20.8)		2226 (26.0)	2215 (25.8)	
**≥75 years**	795 (8.9)	1120 (6.6)		741 (8.6)	798 (9.3)	
**Sex (Male)**	8354 (93.7)	16,134 (94.4)	0.032	8029 (93.7)	8031 (93.7)	0.001
**CHA_2DS_2-VASc**	2.4 (1.6)	1.7 (1.5)	0.284	2.4 (1.6)	2.4 (1.6)	0.003
**0**	846 (9.5)	2405 (14.1)		834 (9.7)	851 (9.9)	
**1**	2084 (23.4)	5339 (31.3)		2050 (23.9)	2150 (25.1)	
**≥2**	5989 (67.2)	9340 (54.7)		5689 (66.4)	5572 (65.0)	
**Hypertension**	6106 (68.5)	10,939 (64.0)	0.093	5834 (68.1)	5843 (68.2)	0.002
**Diabetes mellitus**	2266 (25.4)	4096 (24.0)	0.033	2165 (25.3)	2111 (24.6)	0.014
**Dyslipidemia**	984 (11.0)	2060 (12.1)	0.032	958 (11.2)	959 (11.2)	<0.001
**Heart failure**	2456 (27.5)	3679 (21.5)	0.139	2320 (27.1)	2367 (27.6)	0.012
**Prior MI**	766 (8.6)	850 (5.0)	0.144	686 (8.0)	698 (8.1)	0.005
**PAD**	1761 (19.7)	3195 (18.7)	0.026	1695 (19.8)	1681 (19.6)	0.004

© 2022 Lee HJ et al. *JAMA Network Open*.

**CKD**	1186 (13.3)	1543 (9.0)	0.135	1092 (12.7)	1111 (13.0)	0.006
**COPD**	2059 (23.1)	2990 (17.5)	0.139	1902 (22.2)	1857 (21.7)	0.012
**Cancer**	729 (8.2)	513 (3.0)	0.226	587 (6.9)	505 (5.9)	0.039
**OAC**	3000 (33.6)	3771 (22.1)	0.260	2791 (32.6)	2823 (32.9)	0.008
**Warfarin**	2422 (27.2)	2988 (17.5)	0.233	2251 (26.3)	2295 (26.8)	0.011
**DOAC**	822 (9.2)	1027 (6.0)	0.121	753 (8.8)	753 (8.8)	<0.001
**Aspirin**	1889 (21.2)	3513 (20.6)	0.015	1823 (21.3)	1838 (21.4)	0.004
**$P2Y_{12}$ inhibitor**	838 (9.4)	1079 (6.3)	0.114	780 (9.1)	800 (9.3)	0.008
**Statin**	1789 (20.1)	2690 (15.8)	0.112	1686 (19.7)	1684 (19.6)	<0.001
**BMI (kg/m2)**	24.6 (3.4)	24.4 (3.4)	0.067	24.6 (3.4)	24.6 (3.5)	0.001
**Heavy drinker**	722 (8.1)	2665 (15.6)	0.233	720 (8.4)	674 (7.9)	0.019
**Regular exercise**	2056 (23.1)	3376 (19.8)	0.080	1941 (22.6)	1979 (23.1)	0.010
**Low income**	1500 (16.8)	3057 (17.9)	0.028	1452 (16.9)	1420 (16.6)	0.0100

Continuous variables are presented as mean ± standard deviation and categorical variables are presented as numbers (percentage).

Abbreviation: ASD, absolute standardized difference; BMI, body mass index; CKD, chronic kidney disease; COPD, chronic obstructive pulmonary disease; DOAC, direct oral anticoagulant; MI, myocardial infarction; OAC, oral anticoagulant; PAD, peripheral artery disease; PS, propensity score.

© 2022 Lee HJ et al. *JAMA Network Open*.

eTable 6. Event Number, Incidence Rate, and Hazard Ratio of Dementia After Propensity Score Matching

	Number	Events	IR	HR (95% CI)	p-value
**Total dementia**					0.037
**Current smoker**	8573	344	12.8	1 (reference)	
**Quit-smoker**	8573	298	10.9	0.848 (0.726-0.991)	
**Alzheimer disease**					0.059
**Current smoker**	8573	240	8.9	1 (reference)	
**Quit-smoker**	8573	205	7.5	0.836 (0.694-1.007)	
**Vascular dementia**					0.012
**Current smoker**	8573	77	2.9	1 (reference)	
**Quit-smoker**	8573	50	1.8	0.636 (0.446-0.908)	

IR, per 1000 person-years
Abbreviation: CI, confidence interval; HR, hazard ratio; IR, incidence rate.

© 2022 Lee HJ et al. *JAMA Network Open*.

eTable 7. Competing Risk Analysis

	Model 2<br>HR (95% CI)	p-value	Model 2a<br>HR (95% CI)	p-value
Total dementia		<0.001		<0.001
Never smoker	0.736 (0.663-0.819)		1 (reference)	
Ex-smoker	0.746 (0.673-0.827)		1.013 (0.933-1.100)	
Quit-smoker	0.839 (0.727-0.968)		1.139 (1.003-1.294)	
Current smoker	1 (reference)		1.358 (1.222-1.509)	
Alzheimer disease		<0.001		<0.001
Never smoker	0.777 (0.685-0.881)		1 (reference)	
Ex-smoker	0.761 (0.673-0.861)		0.980 (0.890-1.078)	
Quit-smoker	0.829 (0.698-0.984)		1.067 (0.916-1.242)	
Current smoker	1 (reference)		1.287 (1.135-1.459)	
Vascular dementia		<0.001		<0.001
Never smoker	0.539 (0.425-0.682)		1 (reference)	
Ex-smoker	0.631 (0.504-0.791)		1.171 (0.955-1.436)	
Quit-smoker	0.631 (0.452-0.88)		1.171 (0.862-1.59)	
Current smoker	1 (reference)		1.856 (1.466-2.351)	

Model 2a showed hazard ratio of each group compared to never smoker as a reference group.
Abbreviation: CI, confidence interval; HR, hazard ratio.

© 2022 Lee HJ et al. *JAMA Network Open*.

eFigure 1. Sensitivity Analysis With 1-Year Landmark Analysis: Hazard Ratios of Smoking Status on the Risk of Total Dementia, Alzheimer Disease, and Vascular Dementia

Group	Number	Events	IR	Model 2 HR (95% CI)	Model 2a HR (95% CI)
**Total dementia**				P<0.001	P<0.001
Never smoker	55299	2799	19.4	0.697 (0.618-0.785)	1 (Reference)
Ex-smoker	29139	992	13.4	0.733 (0.652-0.824)	1.052 (0.958-1.156)
Quit-smoker	7783	229	11.4	0.805 (0.684-0.947)	1.156 (1.000-1.336)
Current smoker	14740	405	10.3	1 (Reference)	1.436 (1.273-1.619)
**Alzheimer's dementia**				P<0.001	P<0.001
Never smoker	55299	2135	14.8	0.752 (0.652-0.866)	1 (Reference)
Ex-smoker	29139	728	9.8	0.765 (0.665-0.880)	1.017 (0.913-1.134)
Quit-smoker	7783	162	8.1	0.814 (0.670-0.988)	1.083 (0.913-1.284)
Current smoker	14740	278	7.1	1 (Reference)	1.331 (1.154-1.534)
**Vascular dementia**				P<0.001	P<0.001
Never smoker	55299	419	2.9	0.492 (0.373-0.648)	1 (Reference)
Ex-smoker	29139	174	2.3	0.618 (0.476-0.802)	1.257 (0.993-1.592)
Quit-smoker	7783	34	1.7	0.552 (0.371-0.821)	1.122 (0.770-1.636)
Current smoker	14740	89	2.3	1 (Reference)	2.034 (1.543-2.681)

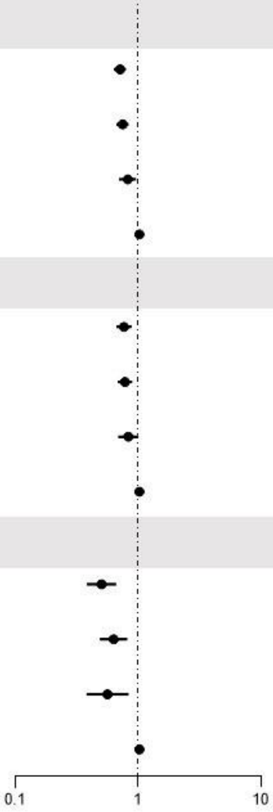

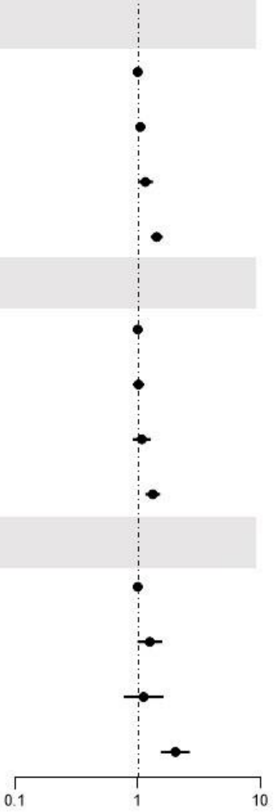

For model 2a, never smokers acted as the reference group. IR, per 1000 person-years. Abbreviation: CI, confidence interval; HR, hazard ratio; IR, incidence rate.

© 2022 Lee HJ et al. *JAMA Network Open*.

eFigure 2. Subgroup Analyses by Sex, Age, CHA₂DS₂-VASc Score, and Previous Stroke (Total Dementia)

(A) Sex

Subgroup		Number	Events	IR	Model 2 HR (95% CI)	Model 2a HR (95% CI)
Total dementia						P_{int}=0.691
Men	Never smoker	19901	845	13.6	0.720 (0.641-0.808)	1 (Reference)
	Ex-smoker	33802	1250	12.1	0.728 (0.654-0.811)	1.011 (0.927-1.104)
	Quit-smoker	8354	269	10.1	0.841 (0.724-0.978)	1.169 (1.017-1.343)
	Current smoker	16134	478	9.1	1 (Reference)	1.389 (1.237-1.560)
Women	Never smoker	45678	2928	20.4	0.683 (0.523-0.891)	1 (Reference)
	Ex-smoker	868	58	23.4	0.785 (0.543-1.135)	1.150 (0.886-1.492)
	Quit-smoker	565	41	23.2	0.726 (0.485-1.089)	1.063 (0.781-1.449)
	Current smoker	950	56	20.1	1 (Reference)	1.464 (1.122-1.911)

Subgroup		Number	Events	IR	Model 2 HR (95% CI)	Model 2a HR (95% CI)
Total Dementia						P_{int}=0.189
<65 years	Never smoker	29023	284	2.9	0.501 (0.376-0.668)	1 (Reference)
	Ex-smoker	17133	128	2.3	0.639 (0.495-0.825)	1.275 (0.958-1.698)
	Quit-smoker	5753	61	3.2	0.863 (0.630-1.180)	1.721 (1.234-2.400)
	Current smoker	12411	115	2.8	1 (Reference)	1.995 (1.497-2.658)
65-74 years	Never smoker	23508	1562	21.0	0.704 (0.602-0.822)	1 (Reference)
	Ex-smoker	11827	519	14.7	0.687 (0.590-0.800)	0.976 (0.857-1.112)
	Quit-smoker	2371	138	18.9	0.797 (0.647-0.982)	1.133 (0.936-1.371)
	Current smoker	3553	252	22.5	1 (Reference)	1.421 (1.216-1.661)
≥ 75 years	Never smoker	13048	1927	57.3	0.786 (0.662-0.934)	1 (Reference)
	Ex-smoker	5710	661	45.2	0.810 (0.682-0.961)	1.030 (0.920-1.153)
	Quit-smoker	795	111	52.5	0.835 (0.656-1.063)	1.062 (0.869-1.298)
	Current smoker	1120	167	57.4	1 (Reference)	1.272 (1.071-1.510)

(C) CHA_2DS_2-VASc

Subgroup		Number	Events	IR	Model 2 HR (95% CI)	Model 2a HR (95% CI)
Total Dementia						P_{int}=0.603
<3	Never smoker	25862	379	4.3	0.661 (0.535-0.817)	1 (Reference)
	Ex-smoker	19450	268	4.2	0.611 (0.501-0.745)	0.924 (0.767-1.113)
	Quit-smoker	5070	72	4.2	0.853 (0.645-1.127)	1.289 (0.982-1.693)
	Current smoker	11828	165	4.1	1 (Reference)	1.512 (1.224-1.868)
≥3	Never smoker	39717	3394	28.9	0.720 (0.638-0.812)	1 (Reference)
	Ex-smoker	15220	1040	24.3	0.755 (0.670-0.852)	1.049 (0.958-1.149)
	Quit-smoker	3849	238	21.0	0.810 (0.687-0.953)	1.125 (0.977-1.295)
	Current smoker	5256	369	24.5	1 (Reference)	1.389 (1.231-1.568)

(D) Prior history of stroke

Subgroup		Number	Events	IR	Model 2 HR (95% CI)	Model 2a HR (95% CI)
Total Dementia						P_{int}=0.058
(-)	Never smoker	17044	591	11.0	0.702 (0.622-0.793)	1 (Reference)
	Ex-smoker	29065	876	9.7	0.721 (0.640-0.812)	1.026 (0.931-1.131)
	Quit-smoker	6856	170	7.7	0.823 (0.695-0.975)	1.172 (1.006-1.364)
	Current smoker	14504	354	7.4	1 (Reference)	1.424 (1.261-1.607)
(+)	Never smoker	2857	254	31.0	0.759 (0.619-0.930)	1 (Reference)
	Ex-smoker	4737	374	28.0	0.742 (0.608-0.905)	0.978 (0.842-1.136)
	Quit-smoker	1498	99	22.1	0.786 (0.609-1.014)	1.036 (0.832-1.290)
	Current smoker	1630	124	26.2	1 (Reference)	1.318 (1.076-1.616)

For model 2a, never smokers acted as the reference group.
IR, per 1000 person-years
Abbreviation: CI, confidence interval; HR, hazard ratio; IR, incidence rate; Pint, p for interaction.

© 2022 Lee HJ et al. *JAMA Network Open*.

eFigure 3. Subgroup Analyses by Sex, Age, CHA_2DS_2-VASc Score, and Previous Stroke (Alzheimer Disease)

(A) Sex

Subgroup		Number	Events	IR	Model 2 HR (95% CI)	Model 2a HR (95% CI)
Alzheimer's dementia						P_{int}=0.749
Men	Never smoker	19901	634	10.2	0.763 (0.665-0.876)	1 (Reference)
	Ex-smoker	33802	907	8.8	0.753 (0.662-0.858)	0.987 (0.891-1.093)
	Quit-smoker	8354	178	6.7	0.812 (0.676-0.976)	1.064 (0.899-1.259)
	Current smoker	16134	325	6.2	1 (Reference)	1.310 (1.141-1.504)
Women	Never smoker	45678	2237	15.6	0.661 (0.488-0.896)	1 (Reference)
	Ex-smoker	868	35	14.1	0.599 (0.383-0.937)	0.906 (0.648-1.266)
	Quit-smoker	565	36	20.3	0.793 (0.508-1.237)	1.199 (0.861-1.668)
	Current smoker	950	43	15.4	1 (Reference)	1.512 (1.116-2.048)

(B) Age

Subgroup		Number	Events	IR	Model 2 HR (95% CI)	Model 2a HR (95% CI)
Alzheimer's dementia						P_{int}=0.519
<65 years	Never smoker	29023	192	2.0	0.512 (0.353-0.742)	1 (Reference)
	Ex-smoker	17133	72	1.3	0.646 (0.459-0.908)	1.262 (0.869-1.832)
	Quit-smoker	5753	33	1.7	0.827 (0.542-1.263)	1.616 (1.041-2.510)
	Current smoker	12411	64	1.6	1 (Reference)	1.954 (1.347-2.833)
65-74 years	Never smoker	23508	1147	15.4	0.748 (0.621-0.901)	1 (Reference)
	Ex-smoker	11827	357	10.1	0.688 (0.572-0.827)	0.919 (0.788-1.073)
	Quit-smoker	2371	99	13.6	0.838 (0.654-1.075)	1.121 (0.895-1.402)
	Current smoker	3553	172	15.4	1 (Reference)	1.337 (1.110-1.610)
≥ 75 years	Never smoker	13048	1532	45.5	0.787 (0.649-0.954)	1 (Reference)
	Ex-smoker	5710	513	35.1	0.800 (0.660-0.971)	1.017 (0.895-1.155)
	Quit-smoker	795	82	38.8	0.773 (0.586-1.019)	0.982 (0.778-1.238)
	Current smoker	1120	132	45.4	1 (Reference)	1.271 (1.048-1.541)

(C) CHA_2DS_2-VASc

Subgroup		Number	Events	IR	Model 2 HR (95% CI)	Model 2a HR (95% CI)
Alzheimer's dementia						P_{int}=0.446
<3	Never smoker	25862	271	3.1	0.711 (0.550-0.919)	1 (Reference)
	Ex-smoker	19450	174	2.7	0.583 (0.456-0.746)	0.820 (0.656-1.026)
	Quit-smoker	5070	48	2.8	0.865 (0.613-1.220)	1.217 (0.875-1.692)
	Current smoker	11828	106	2.6	1 (Reference)	1.407 (1.089-1.818)
≥3	Never smoker	39717	2600	22.1	0.750 (0.651-0.864)	1 (Reference)
	Ex-smoker	15220	768	17.9	0.774 (0.671-0.892)	1.032 (0.929-1.146)
	Quit-smoker	3849	166	14.6	0.789 (0.649-0.960)	1.053 (0.891-1.245)
	Current smoker	5256	262	17.4	1 (Reference)	1.334 (1.157-1.537)

(D) Prior history of stroke

Subgroup		Number	Events	IR	Model 2 HR (95% CI)	Model 2a HR (95% CI)
Alzheimer's dementia						P_{int}=0.0849
(-)	Never smoker	56985	2197	12.2	0.746 (0.646-0.862)	1 (Reference)
	Ex-smoker	29821	669	7.2	0.739 (0.641-0.852)	0.990 (0.884-1.108)
	Quit-smoker	7324	148	6.3	0.846 (0.692-1.034)	1.133 (0.948-1.353)
	Current smoker	15366	275	5.5	1 (Reference)	1.340 (1.160-1.547)
(+)	Never smoker	8594	674	27.2	0.772 (0.606-0.985)	1 (Reference)
	Ex-smoker	4849	273	19.8	0.733 (0.577-0.930)	0.948 (0.795-1.131)
	Quit-smoker	1595	66	13.8	0.702 (0.511-0.963)	0.908 (0.692-1.193)
	Current smoker	1718	93	18.7	1 (Reference)	1.295 (1.016-1.650)

For model 2a, never smokers acted as the reference group.

IR, per 1000 person-years

Abbreviation: CI, confidence interval; HR, hazard ratio; IR, incidence rate; Pint, p for interaction.

© 2022 Lee HJ et al. *JAMA Network Open*.

eFigure 4. Subgroup Analyses by Sex, Age, CHA_2DS_2-VASc Score, and Previous Stroke (Vascular Dementia)

(A) Sex

Subgroup		Number	Events	IR	Model 2 HR (95% CI)	Model 2a HR (95% CI)
Vascular dementia						P_{int}=0.108
Men	Never smoker	19901	123	2.0	0.518 (0.396-0.677)	1 (Reference)
	Ex-smoker	33802	216	2.1	0.605 (0.477-0.768)	1.169 (0.936-1.460)
	Quit-smoker	8354	49	1.8	0.670 (0.477-0.942)	1.294 (0.925-1.811)
	Current smoker	16134	109	2.1	1 (Reference)	1.931 (1.477-2.523)
Women	Never smoker	45678	430	3.0	0.667 (0.342-1.303)	1 (Reference)
	Ex-smoker	868	13	5.2	1.199 (0.510-2.817)	1.797 (1.033-3.125)
	Quit-smoker	565	2	1.1	0.249 (0.054-1.157)	0.373 (0.093-1.499)
	Current smoker	950	9	3.2	1 (Reference)	1.499 (0.767-2.927)

(B) Age

Subgroup		Number	Events	IR	Model 2 HR (95% CI)	Model 2a HR (95% CI)
Vascular dementia						P_{int}=0.297
<65 years	Never smoker	29023	62	0.6	0.540 (0.320-0.910)	1 (Reference)
	Ex-smoker	17133	40	0.7	0.583 (0.371-0.916)	1.080 (0.643-1.811)
	Quit-smoker	5753	16	0.8	0.673 (0.373-1.216)	1.247 (0.659-2.361)
	Current smoker	12411	38	0.9	1 (Reference)	1.852 (1.099-3.121)
65-74 years	Never smoker	23508	259	3.5	0.494 (0.346-0.706)	1 (Reference)
	Ex-smoker	11827	106	3.0	0.658 (0.472-0.917)	1.332 (0.975-1.820)
	Quit-smoker	2371	16	2.2	0.422 (0.241-0.739)	0.854 (0.497-1.467)
	Current smoker	3553	54	4.8	1 (Reference)	2.023 (1.416-2.891)
≥ 75 years	Never smoker	13048	232	6.9	0.581 (0.372-0.906)	1 (Reference)
	Ex-smoker	5710	83	5.7	0.616 (0.395-0.961)	1.061 (0.771-1.459)
	Quit-smoker	795	19	9.0	0.916 (0.505-1.659)	1.577 (0.960-2.591)
	Current smoker	1120	26	9.0	1 (Reference)	1.723 (1.103-2.689)

(C) CHA_2DS_2-VASc

Subgroup		Number	Events	IR	Model 2 HR (95% CI)	Model 2a HR (95% CI)
Vascular dementia						P_{int}=0.864
<3	Never smoker	25862	66	0.8	0.510 (0.322-0.806)	1 (Reference)
	Ex-smoker	19450	58	0.9	0.578 (0.386-0.867)	1.134 (0.741-1.737)
	Quit-smoker	5070	14	0.8	0.673 (0.366-1.237)	1.320 (0.705-2.471)
	Current smoker	11828	43	1.1	1 (Reference)	1.961 (1.240-3.102)
≥3	Never smoker	39717	487	4.1	0.533 (0.401-0.708)	1 (Reference)
	Ex-smoker	15220	171	4.0	0.635 (0.482-0.837)	1.191 (0.944-1.502)
	Quit-smoker	3849	37	3.2	0.607 (0.409-0.901)	1.138 (0.793-1.634)
	Current smoker	5256	75	5.0	1 (Reference)	1.876 (1.412-2.491)

(D) Prior history of stroke

Subgroup		Number	Events	IR	Model 2 HR (95% CI)	Model 2a HR (95% CI)
Vascular dementia						P_{int}=0.775
(-)	Never smoker	56985	386	2.2	0.462 (0.348-0.614)	1 (Reference)
	Ex-smoker	29821	149	1.6	0.570 (0.436-0.745)	1.234 (0.957-1.590)
	Quit-smoker	7324	29	1.2	0.564 (0.370-0.859)	1.220 (0.813-1.833)
	Current smoker	15366	89	1.8	1 (Reference)	2.165 (1.629-2.877)
(+)	Never smoker	8594	167	6.7	0.726 (0.464-1.138)	1 (Reference)
	Ex-smoker	4849	80	5.8	0.776 (0.504-1.194)	1.068 (0.761-1.500)
	Quit-smoker	1595	22	4.6	0.753 (0.432-1.312)	1.036 (0.635-1.690)
	Current smoker	1718	29	5.8	1 (Reference)	1.377 (0.878-2.157)

For model 2a, never smokers acted as the reference group.

IR, per 1000 person-years

Abbreviation: CI, confidence interval; HR, hazard ratio; IR, incidence rate; Pint, p for interaction.

© 2022 Lee HJ et al. *JAMA Network Open*.

eReferences

1. Choi EK. Cardiovascular Research Using the Korean National Health Information Database. Korean Circ J. 2020;50:e90. https://doi.org/10.4070/kcj.2020.0171.

2. Cheol Seong S, Kim YY, Khang YH, et al. Data Resource Profile: The National Health Information Database of the National Health Insurance Service in South Korea. Int J Epidemiol. 2017;46:799-800.

3. Lee SR, Choi EK, Han KD, Cha MJ, Oh S. Trends in the incidence and prevalence of atrial fibrillation and estimated thromboembolic risk using the CHA_2DS_2-VASc score in the entire Korean population. Int J Cardiol. 2017;236:226-31.

4. Kang SH, Choi EK, Han KD, et al. Underweight is a risk factor for atrial fibrillation: A nationwide population-based study. Int J Cardiol. 2016;215:449-56.

5. Lee SR, Choi EK, Park CS, et al. Direct Oral Anticoagulants in Patients With Nonvalvular Atrial Fibrillation and Low Body Weight. J Am Coll Cardiol. 2019;73:919-31.

6. Lee SR, Choi EK, Kwon S, et al. Effectiveness and Safety of Contemporary Oral Anticoagulants Among Asians With Nonvalvular Atrial Fibrillation. Stroke. 2019;50:2245-9.

7. Lip GY, Nieuwlaat R, Pisters R, Lane DA, Crijns HJ. Refining clinical risk stratification for predicting stroke and thromboembolism in atrial fibrillation using a novel risk factor-based approach: the euro heart survey on atrial fibrillation. Chest. 2010;137:263-72.

© 2022 Lee HJ et al. *JAMA Network Open*.
